# Efficacy of decitabine-loaded gelatinases-stimuli nanoparticles in overcoming cancer drug resistance is mediated via its enhanced demethylating activity to transcription factor AP-2 epsilon

**DOI:** 10.18632/oncotarget.21274

**Published:** 2017-09-26

**Authors:** Yi-Dong Hong, Jian Zhang, Ming Zhuang, Wei Li, Puy-Uan Wu, Ru-Tian Li, Nan Hu, Bao-Xiang Bian, Zi-Yan Song, Feng-Lei Wu

**Affiliations:** ^1^ Department of Oncology, Affiliated Lianyungang Hospital of Xuzhou Medical University, Lianyungang, Jiangsu, 221002, China; ^2^ Deparment of Oncology, School of Medicine, Jiangsu University, Zhengjiang, Jiangsu, 212013, China; ^3^ Department of General Surgery, Huai'an First People’s Hospital, Nanjing Medical University, Huai'an, Jiangsu, 223000, China; ^4^ Center of Research Laboratory, Affiliated Lianyungang Hospital of Xuzhou Medical University, Lianyungang, Jiangsu, 222000, China; ^5^ The Comprehensive Cancer Centre of Drum Tower Hospital, Medical School of Nanjing University, Clinical Cancer Institute of Nanjing University, Nanjing, Jiangsu, 210008, China

**Keywords:** TFAP2E, hypermethylation, epigenetic drugs, nanoparticles, drug delivery

## Abstract

Hypermethylation of the transcription factor AP-2 epsilon (TFAP2E) gene affects 5-fluorouridine (5-FU) resistance in gastric cancer (GC) patients. The epigenetic inhibitor 5-Aza-2′-deoxycytidine (DAC), which reverses DNA methylation by targeting DNA methyltransferases (DNMTs), has potential to sensitize GC to 5-FU. Nevertheless, DNA demethylation only DAC transiently occurs since DAC is unstable in aqueous solutions, which limits its potential. Here we developed intelligent nanoparticles (NPs) comprising gelatinase with polyethylene glycol (PEG) and poly-ε-caprolactone) (PCL) to specifically deliver DAC (DAC-TNPs) to tumors. DAC-carrying PEG-PCL NPs (DAC-NPs) lacking gelatinase features served as controls. 72 hours after administration of DAC-TNPs or DAC-NPs, 5-FU was sequentially applied to GC cells and human GC xenografts in nude mice. Both *in vitro* and *in vivo* evaluations demonstrated that the combination treatment of DAC-TNPs and 5-FU greatly improved tumor suppression in GC cells and mouse xenograft models with hypermethylation TFAP2E (MKN45 cells). We thus propose that the sequential administration of DAC-TNPs and 5-FU could be significant in the development of novel targeted therapies.

## INTRODUCTION

Gastric cancer (GC) ranks 4th in incidence among malignancies, but constitutes the 3rd deadliest cancer worldwide [1]. Most GC patients are asymptomatic in the early stage, and present with advanced disease by the time of diagnosis [2]. Advanced GC is often treated with chemotherapeutics, with 5-FU included in standard regimens. Unfortunately, individuals with GC respond differently to 5-FU therapy, whose effectiveness is hampered by intrinsic and acquired resistance. Accordingly, new efficient drug combinations are constantly explored.

Abnormal gene methylation, an epigenetic modification, regulates the expression of several genes, changing patient sensitivity to chemotherapeutics. For instance, hypermethylation of the transcription factor AP-2 epsilon (TFAP2E) affects the therapeutic efficacy of 5-FU based chemotherapy in colorectal cancer [3] and GC [4]. The hypermethylation decreases TFAP2E expression and consequently increases expression of its downstream target dickkopf homolog 4 protein (DKK4), which is related to 5-FU resistance [3, 4]. 5-Aza-2′-deoxycytidine (DAC) increases TFAP2E levels in malignancies via DNA demethylation, increasing sensitivity towards 5-FU [3, 4]. DAC is currently used for myelodysplastic syndrome (MDS) and acute myeloid leukemia (AML) [5]. It is also efficacious in solid tumors, including lung cancer, esophageal cancer, and pleural mesothelioma [6]. However, the efficacy of DAC is limited due to its instability, with a 10–35 minute half-life *in vivo* [7].

The properties of nanoparticles (NPs) could help deliver drugs to target sites through enhanced permeability and retention (EPR) [8]. Thus, NPs could effectively deliver anticancer agents. Many reports have confirmed the superiority of NPs, e.g. the enhanced efficacy of docetaxel-loaded nanoparticles [9] and decitabine-loaded nanogels [10]. Polyethylene glycol (PEG) and poly-ε-caprolactone (PCL), with biodegradability and biocompatibility, are FDA approved and currently applied in the biomedical field [11, 12]. In our previous work, a gelatinase based drug delivery system was designed. The cancer-specific gelatinase cleavable peptide was inserted between PEG and PCL to make it cleavable since gelatinases abound in most tumors [13]. NPs have been systematically assessed for their use in cancer therapy [9, 14, 15]. NPs with the gelatinase-cleavable peptide are more efficient compared with their counterparts without, showing improved intracellular uptake, increased accumulation in tumors, and long-term retention [9]

In our previous study, we demonstrated that 5-FU and DAC encapsulated with gelatinase-stimuli NPs effectively suppress MKN45 cell proliferation [15]. However, the synergistic effect of NPs-5-FU-DAC was not obviously observed before 72 hours. One potential reason is that DAC exerts its demethylation effects during cell replication; therefore, several cell replication rounds are required before such synergistic effects can be observed. In this study, MKN45 cell treatment with DAC-loaded NPs or DAC-loaded NPs was performed for 72 hours, followed by 5-FU application. We found that the sequential application of 5-FU after DAC-TNPs results in improved therapeutic efficacy in GC cancer.

## RESULTS

### Nanoparticle preparation and characterization

**Figure 1:**
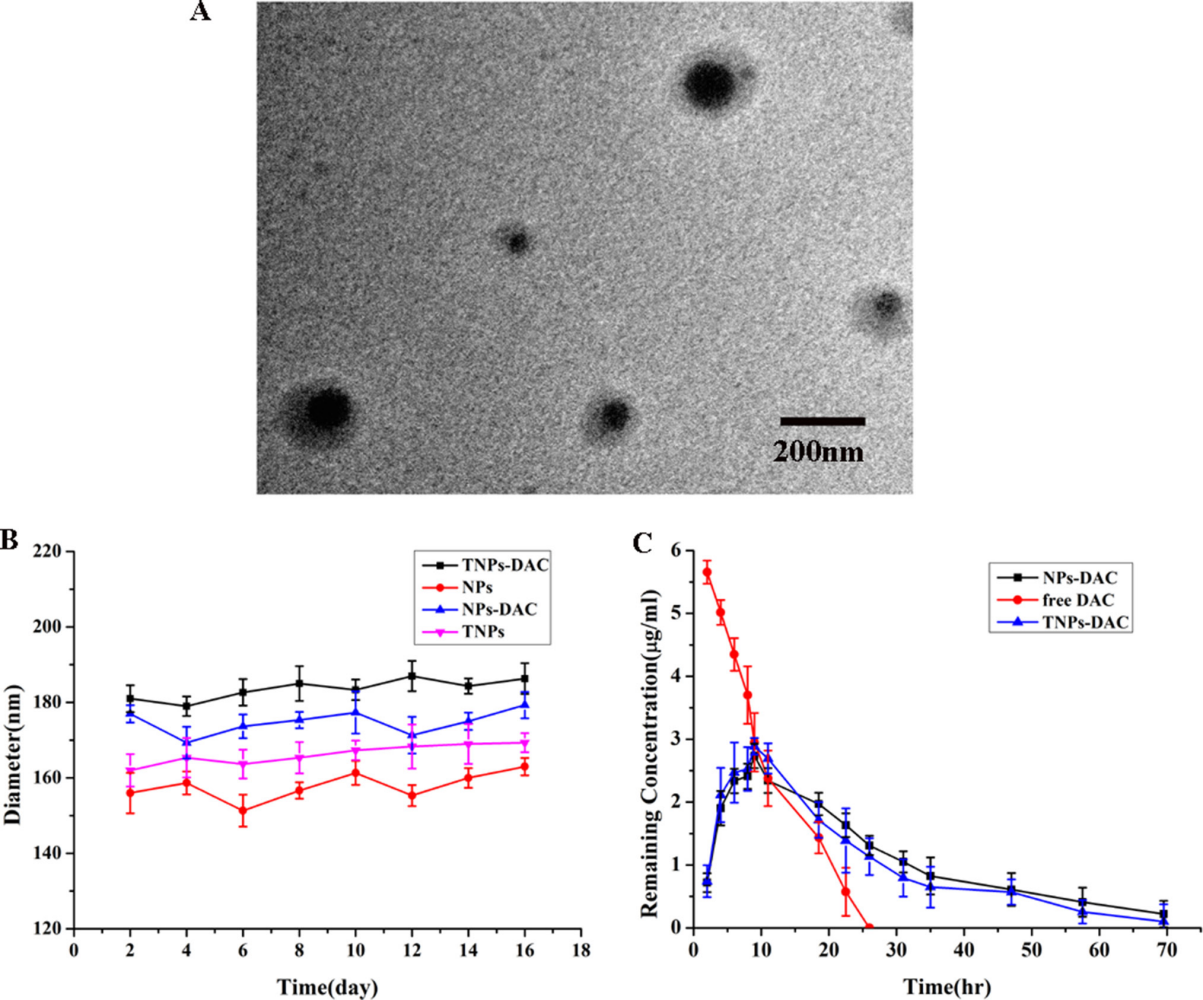
Physicochemical properties of NPs (**A**) Ultrastructure of DAC-TNPs obtained by TEM; (**B**) Stability of NPs. TNP diameters were obtained by DLS (mean ± SD); (**C**) changes of DAC’s remnant of various groups. NPs, nanoparticles; TNPs, Gelatinases-stimuli nanoparticles; TEM, transmission electron microscopy; DAC, 5-Aza-2′-deoxycytidine; DLS, dynamic light scattering; SD, standard deviation.

DAC-NP and DAC-TNP preparation was performed with the double-emulsion solvent evaporation method; Control nanoparticles had no drug included. The average nanoparticle size ranged from 176.3 to 196.3 nm as measured by TEM, which could facilitate DAC delivery by the EPR effect [8]. This finding indicated that the nanoparticles were homogeneous in size since they were all approximately 190 nm (Figure 1A). The NP sizes were unchanged after 16 days (Figure 1B). The polydispersity ranged from 0.105 to 0.202; average zeta potential ranged between −7.97 and −11.23 mV. The efficiencies of DAC loading in DAC-NPs and DAC-TNPs were 1.623 ± 0.22% and 1.723 ± 0.21%, respectively; encapsulation efficiencies for DAC were 61.4 ± 2.31% and 67.4 ± 1.91%, respectively (Table 1).

**Table 1:** Mean particle size and drug load efficiency of two kinds of nanoparticles

Nanoparticles	Diameters (nm)^a^	Polydispersitya	zeta potentiala	DLC (%)^b^	EE (%)^c^
NPs	176.3 ± 5.6	0.105 ± 0.050	−8.90 ± 1.17	—	—
TNPs	185.0 ± 7.5	0.208 ± 0.018	−7.97 ± 0.91	—	—
DAC-NPs	191.3 ± 5.9	0.147 ± 0.019	−8.94 ± 1.19	61.4 ± 2.31	1.623 ± 0.22
DAC-TNPs	196.3 ± 8.7	0.202 ± 0.058	−11.23 ± 1.05	67.4 ± 1.91	1.723 ± 0.21

^a^ The SD value was for the mean particle size obtained from the three measurements.

^b^ DLC, drug loading content.

^c^ EE, encapsulation efficiency.

### *In vitro* DAC degradation from DAC-NPs and DAC-TNPs

The concentration of free DAC in PBS quickly decreased within 24 h. However DAC in NPs and TNPs degraded at a reduced rate compared with free DAC (72 h) (Figure 1C), indicating that the half-life of DAC was markedly prolonged after encapsulation in nanoparticles. Therefore, nanoparticles carrying DAC could be more useful in gene demethylation.

### Immunohistochemical and zymography analyses of GC cells

**Figure 2:**
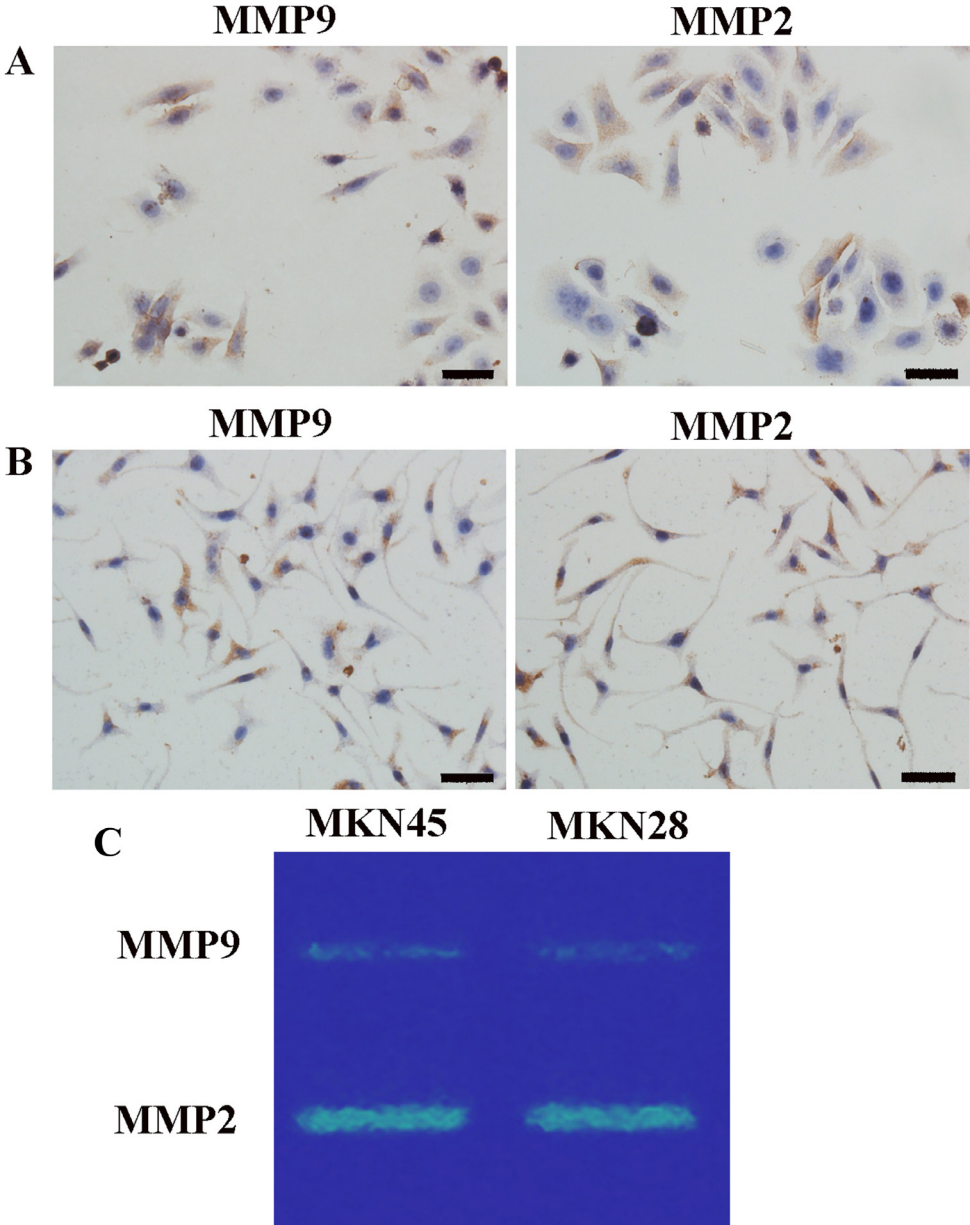
Gelatinase (MMP2/9) levels Immunohistochemistry for gelatinase (MMP2/9) protein levels in MKN45 (**A**) and MKN28 (**B**) cells (×400); (**C**) MMP2 and MMP9 amounts in GC cells assessed by zymography; comparable amounts of gelatinase were found in MKN45 and MKN28 cells. MMP2 and MMP9 were localized to the cytoplasm and cell membrane, with no nuclear expression. High MMP2 amounts were detected in GC cells. MMP9 expression levels were somewhat lower compared with MMP2 levels in both GC cell lines. Scale bar, 50 μm. (MMP, matrix metalloproteinases).

Matrix metalloproteinase 2 (MMP)2 and MMP9 protein levels in MKN45 and MKN28 cells were determined by immunohistochemistry (IHC). Expression was reflected by brown cytoplasmic signals (Figure 2A, 2B). We confirmed the findings by zymography analysis. In agreement with IHC, zymography demonstrated that both cell lines had comparable amounts of MMP2 and MMP9 (Figure 2C).

### Nanoparticle uptake by gastric cancer cells

**Figure 3:**
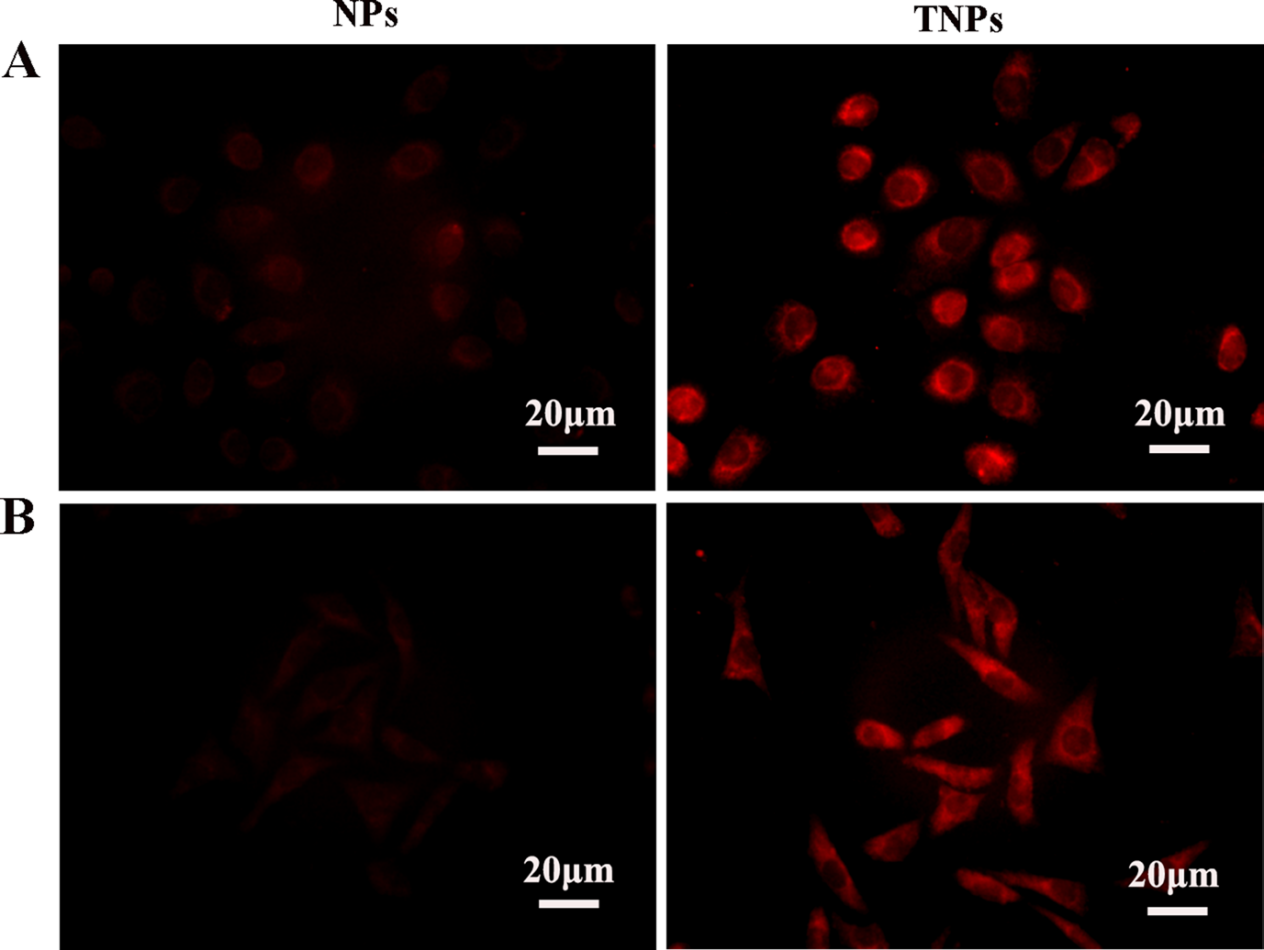
MKN45 (**A**) and MKN28 (**B**) cells were treated with Rhodamine-B-loaded NPs and TNPs, respectively. Fluorescent signals were mostly in the cytoplasm, and only minimal signals in nuclei. TNPs showed more pronounced signals than NPs. Scale bar, 20 μm.

GC cells were treated with Rhodamine B-loaded NPs and TNPs for 2 h. Cellular internalization was investigated by detecting red fluorescence in the cytoplasm. Red fluorescence was found in the cytoplasm of both MKN45 and MKN28 cells, while nuclear signals were low (Figure 3), indicating that drug-encapsulated nanoparticles were readily taken up by the cells, with cytoplasmic accumulation. In both MKN45 (Figure 3A) and MKN28 (Figure 3B) cells, the fluorescence intensity of TNPs was stronger than that of NPs, suggesting effective targeting of TNPs into GC cells.

### *In vitro* study of sequential delivery DAC-TNPs and 5-FU

**Figure 4:**
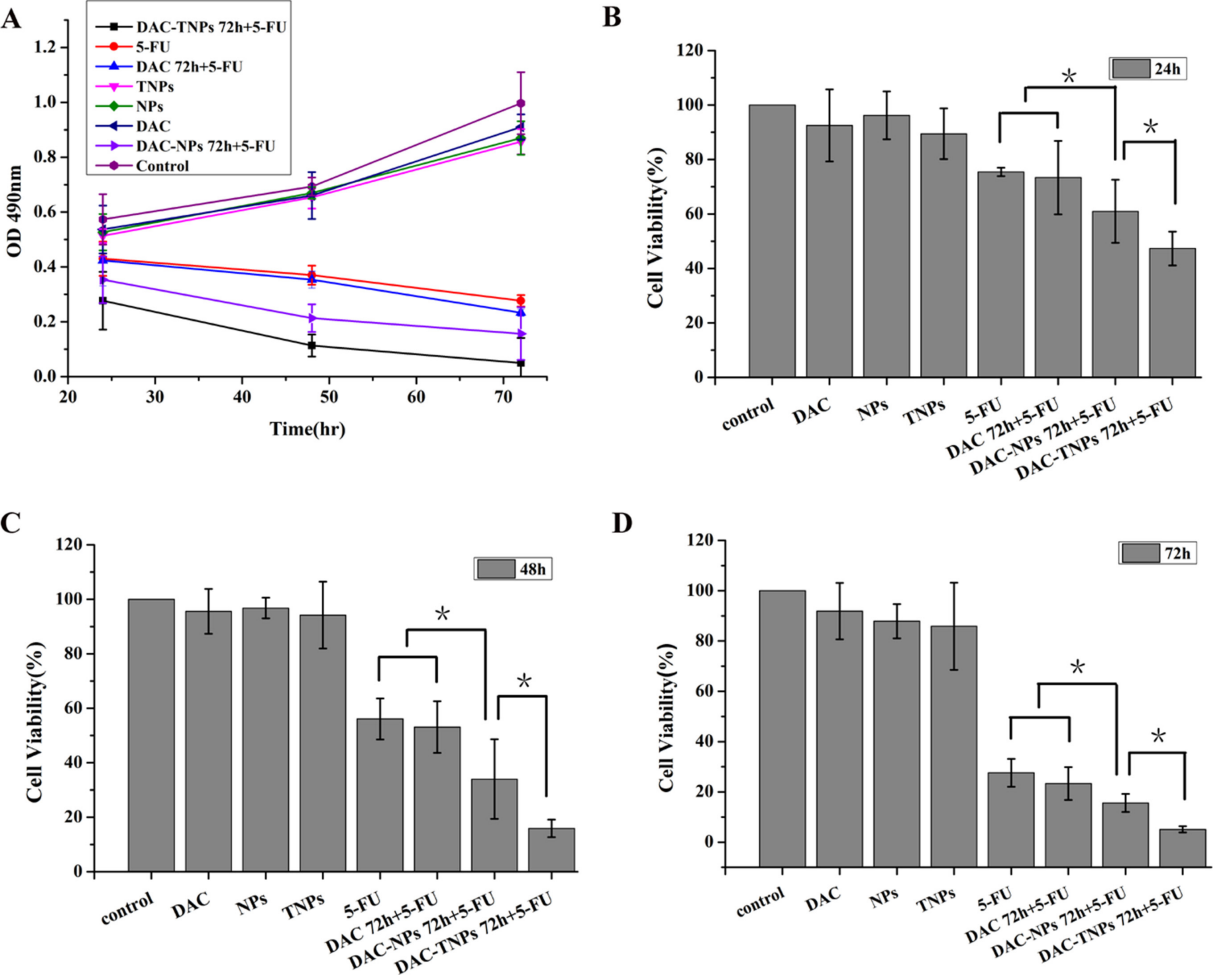
Effects of 5-FU, DAC, and nanoparticles on MKN45 cell viability Cell viability was assessed by the MTT every 24h for 3 days. Saline was used as treatment control (**A**). NPs and TNPs (no drug), and free DAC mildly reduced cell viability. Sequential administration of free DAC and 5-FU showed a similar suppression effects with 5-FU alone (*P* > 0.05). However, sequential administration of DAC encapsulated in different nanoparticles and 5-FU exhibited stronger suppressive effects compared with the other groups; the inhibitory effects of DAC-TNPs 72h + 5-FU were more pronounced than those of DAC-NPs 72h + 5-FU (^*^P < 0.05) after the treatment of 24h (**B**), 48h (**C**) and 72h (**D**). DAC, 5-Aza-2′-deoxycytidine; NPs, nanoparticles; TNPs, gelatinases-stimuli nanoparticles; 5-FU, 5-fluorouracil.

**Figure 5:**
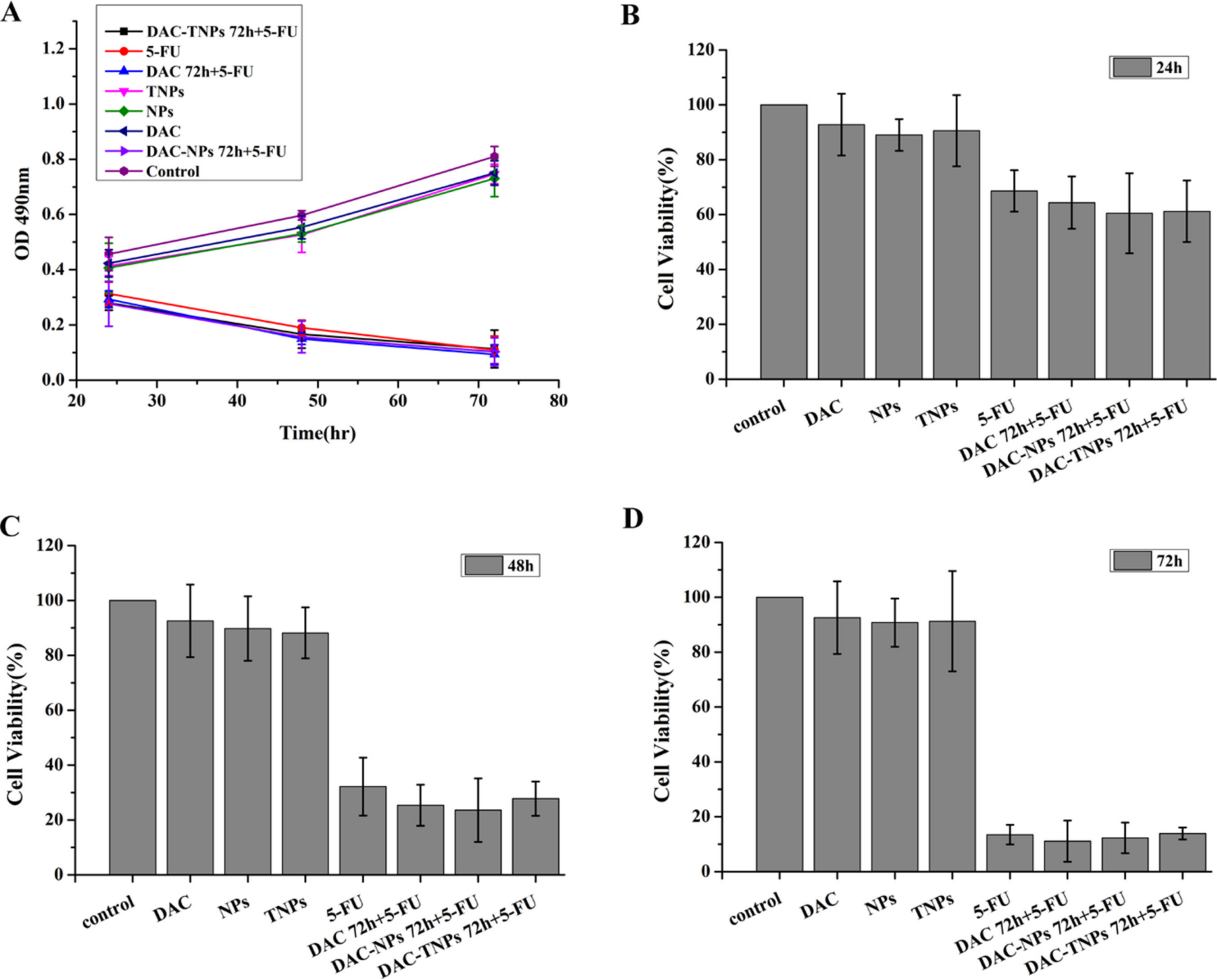
Effects of 5-FU, DAC, and nanoparticles on MKN28 cell viability Cell viability was assessed by the MTT every 24h for 3 days. Saline was used as treatment control (**A**). Control NPs and TNPs (no drug), and free DAC mildly reduced cell viability. Sequential administration of free DAC and 5-FU showed a similar suppression effects with 5-FU alone (*P* > 0.05). Sequential treatment with DAC encapsulated in different nanoparticles and 5-FU exhibited similar suppressive effects compared with both 5-FU and DAC 72h + 5-FU (*P* > 0.05) after the treatment of 24h (**B**), 48h (**C**) and 72h (**D**). DAC, 5-Aza-2′-deoxycytidine; NPs, nanoparticles; TNPs, gelatinases-stimuli nanoparticles; 5-FU, 5-fluorouracil.

Figure 4A (MKN45) and Figure 5A (MKN28) showed the cytotoxicity of NPs, TNPs, DAC, 5-FU, DAC 72 h + 5-FU, DAC-NPs 72 h + 5-FU and DAC-TNPs 72 h + 5-FU. We found that incubation with NPs, TNPs and DAC resulted in mild cell cytotoxicity. Cell cytotoxicity levels of 5-FU and DAC 72 h + 5-FU were not significantly different in both cell lines (*P* > 0.05, Figures 4 and 5). In MKN45 cells, the rate of DAC-NPs 72 h + 5-FU was higher than those of 5-FU and DAC 72 h + 5-FU (*P* < 0.05, Figure 4); the DAC-TNPs 72 h + 5-FU group had the highest inhibition rates in all groups. However, in MKN28 cells, similar inhibition rates in the 5-FU, DAC 72 h + 5-FU, DAC-NPs 72 h + 5-FU and DAC-TNPs 72 h + 5-FU groups were observed. (*P* > 0.05, Figure 5).

### The sequential application of DAC-TNPs and 5-FU improves the antitumor effect *in vivo*

**Figure 6:**
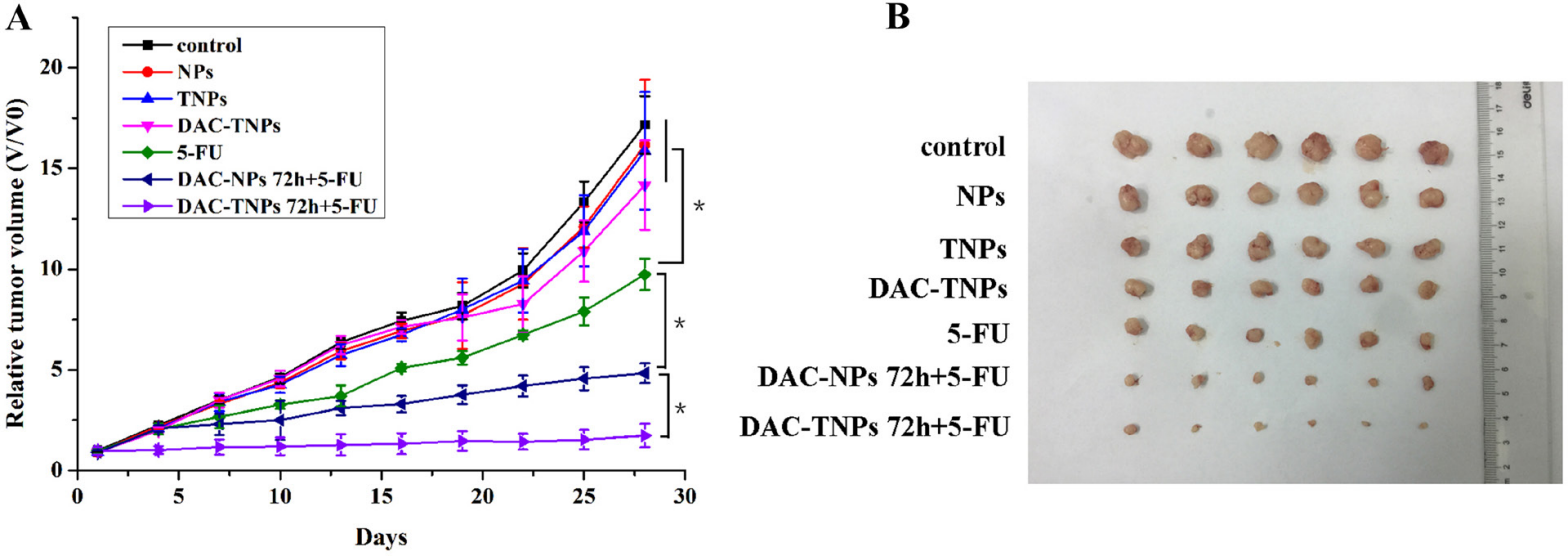
Antitumor efficacies of different treatments in nude mouse MKN45 xenograft models (**A**) Relative tumor volumes. (**B**) Tumor images after the treatment. (^*^P < 0.05)

Blank NPs, TNPs, DAC-TNPs did not inhibit tumor growth. Tumors treated with 5-FU, DAC-NPs+5-FU and DAC-TNPs+5-FU were markedly inhibited in comparison with those treated with saline (*P* < 0.01); the differences among the three groups were not significant during the first 7 days of treatment (*P* > 0.05). DAC-NPs 72 h+5-FU and DAC-TNPs 72 h+5-FU began to show greater antitumor effects in comparison with other test articles in the first 7 days, with anticancer advantages becoming increasingly pronounced with time (*P* < 0.05). Interestingly, DAC-TNPs 72 h+5-FU completely halted tumor growth, with overtly superior outcome compared with DAC-NPs 72 h+5-FU (*P* < 0.05). Tumors treated with DAC-TNPs 72 h+5-FU were the most reduced in size (*P* < 0.05) (Figure 6).

### TFPA2E and DKK4 levels after treatment with DAC-NPs and DAC-TNPs

**Figure 7:**
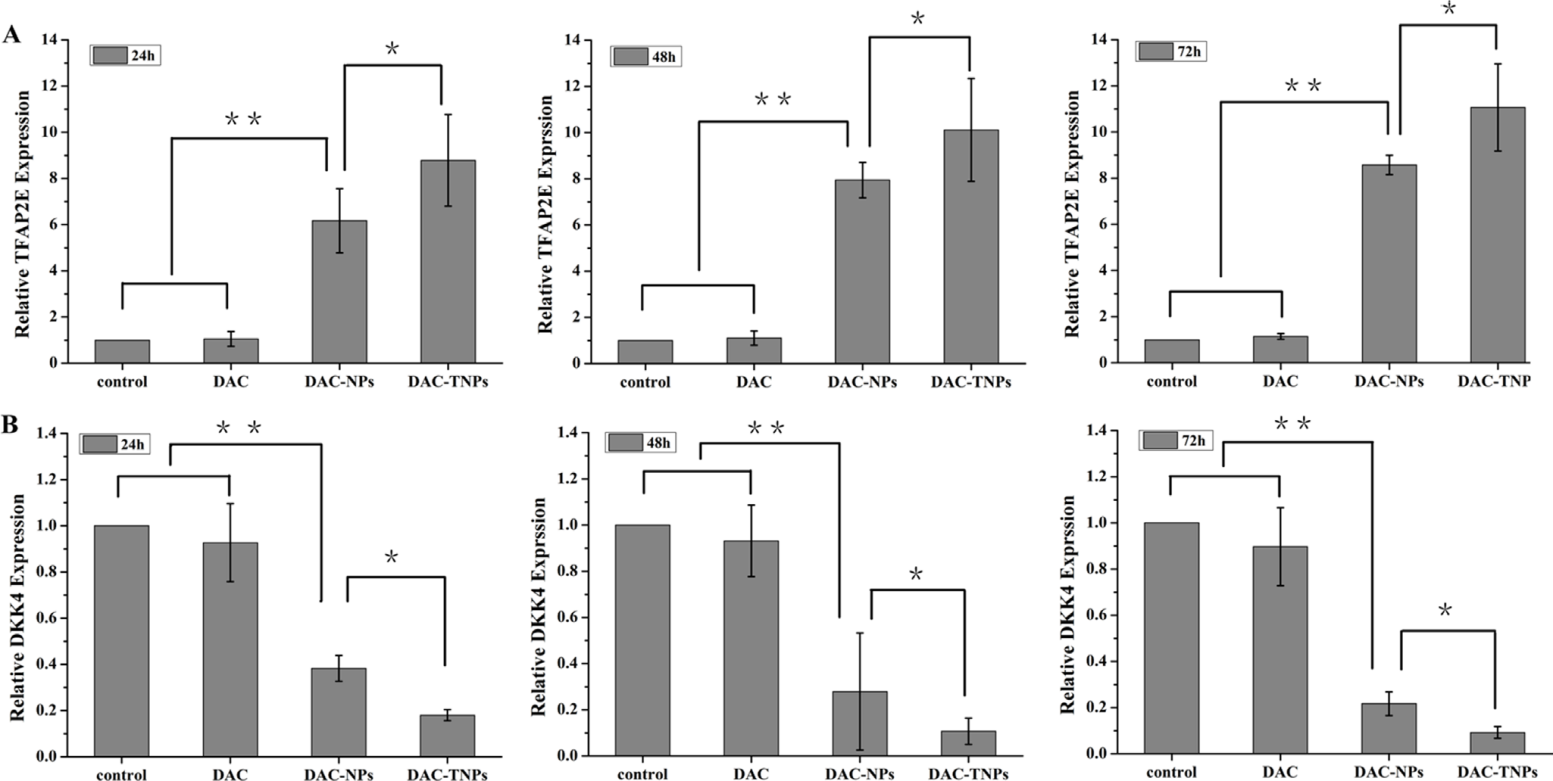
TFAP2E and DKK4 mRNA levels in MKN45 cells after treatment with DAC, DAC-NPs, and DAC-TNPs, respectively, at 24, 48 and 72 h TFAP2E upregulation was most pronounced after treatment with DAC-TNPs, followed by DAC-NPs, and DAC. Compared with saline treated controls, DAC-NPs and DAC-TNPs upregulated TFAP2E (**A**) ^**^P < 0.01), while downregulating the downstream DKK4 (**B**) ^**^P < 0.01). The expression levels of TFAP2E in the DAC-TNPs group were markedly increased at 72 h in comparison with those of the DAC-NPs group (A, ^*^P < 0.05), with DKK4 decreasing accordingly (B, ^*^P < 0.05). TFAP2E and DKK4 amounts were unchanged after treatment with free DAC (10 μmol/L) (A and B, *P* > 0.05). DAC, 5-Aza-2′-deoxycytidine; NPs, nanoparticles; NPs, gelatinases-stimuli nanoparticles; 5-FU, 5-fluorouracil; TFAP2E, transcription factor AP-2 epsilon.

**Figure 8:**
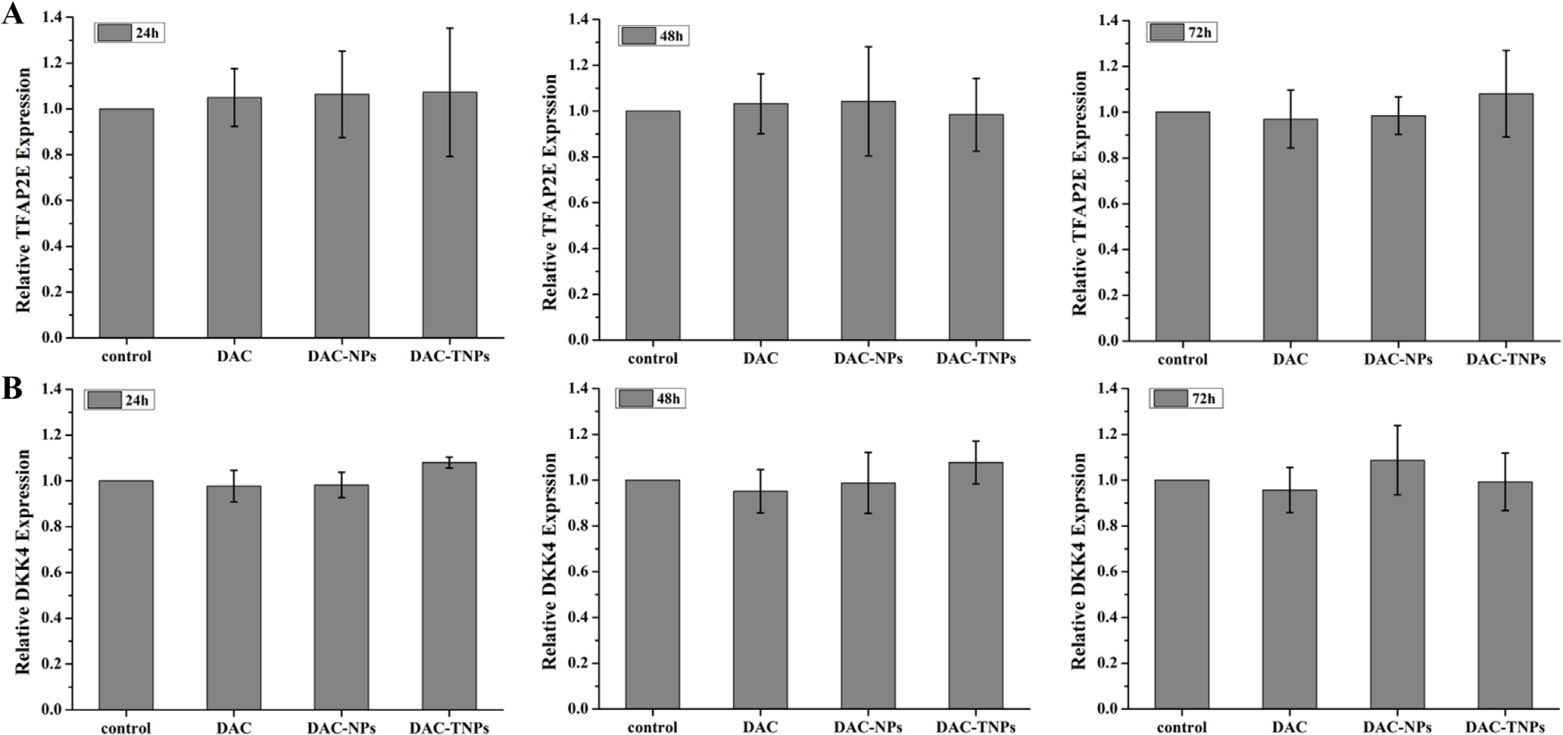
TFAP2E and DKK4 mRNA levels in MKN28 cells after treatment with DAC, DAC-NPs, and DAC-TNPs at various time points (24, 48 and 72 h) In comparison with saline control group, MKN28 cells had similar TFAP2E and DKK4 amounts at all time points (**A** and **B**) *P* > 0.05). DAC, 5-Aza-2′-deoxycytidine; NPs, nanoparticles; NPs, gelatinases-stimuli nanoparticles; 5-FU, 5-fluorouracil; TFAP2E, transcription factor AP-2 epsilon.

DAC, DAC-NPs and DAC-TNPs were assessed for their effects on TFAP2E and the downstream target DKK4, every 24 h in MKN45 and MKN28 cells. Compared with saline controls, treatment with NPs-DAC (10 μmol/L DAC eq) and TNPs-DAC (10 μmol/L DAC eq) resulted in TFAP2E upregulation in the MKN45 cell line (*P* < 0.01), concomitant to DKK4 downregulation (*P* < 0.01). Additionally, TFAP2E levels after treatment with DAC-TNPs were significantly increased compared with those of the DAC-NPs group (*P* < 0.05), with lower levels of DKK4 accordingly (*P* < 0.05). Nevertheless, TFAP2E and DKK4 amounts remained unchanged upon treatment with free DAC at 10 μmol/L (Figure 7). In addition, MKN28 cells showed no significant differences in TFAP2E and DKK4 amounts among various groups (*P* > 0.05, Figure 8).

## DISCUSSION

In our previous study, gelatinase-based nanoparticles were shown to efficiently deliver both epigenetic drugs and chemical agents into gastric cancer cells. However, this combinatory treatment enhances growth suppression of MKN45 after 72 h [15]. Therefore, in this study, we improved the application method with sequential treatments with DAC-TNPs and 5-FU. This treatment setting significantly suppressed the growth of MKN45 cells compared with other groups, upregulating TFAP2E and downregulating DKK4. Notably, these effects were only observed in MKN45 cells with hypermethylated TFAP2E but not in MKN28 cells, probably because TFAP2E levels were not obviously increased after treatment; indeed, TFAP2E methylation in MKN28 cells was not detected.

Nanoparticle drug delivery systems attracts increasing attention owing to their targeted drug delivery potential, which can reduce the side effects of otherwise very toxic drugs. In a previous study, we designed NPs by inserting a gelatinase-cleavage peptide between PEG and PCL for targeted drug delivery in cancer therapy [9]. Through immunohistochemical and gelatin zymography assays, the current study demonstrated that MKN45 and MKN28 cells showed similar gelatinase expression levels (Figure 2A–2C). Meanwhile, Rhodamine B-carrying TNPs in MKN45 cells showed strong signals comparable to those of MKN28 cells, which were both stronger than the signals obtained for Rhodamine B-carrying NPs (Figure 3). These findings corroborate our previously reported data demonstrating that gelatinase-NPs have superior cell uptake compared with non-targeted NPs in tumors with high expression levels of gelatinase [9]. This enhanced uptake of tumor targeting is critical to the treatment of malignancies.

DNA methylation dysregulation has been implicated in resistance to chemotherapeutic drugs [16]. In contrast to genetic mutations, epimutations can be reversed by some interventions such as applying epigenetic drugs. DAC is a popular epigenetic drug that can reactivate genes silenced due to abnormal DNA methylation, approved by the US Food and Drug Administration (FDA) for the treatment of MDS and AML. The anticancer effects of DAC in hematological malignancies have indicated its potential use in solid tumors. Many studies have reported that DAC can sensitize tumor cells to conventional chemotherapeutic drugs in solid tumors. Indeed, DAC restores sensitivity of colorectal cancer SW48 cells to 5-FU [17]. In addition, DAC sensitizes human colon cancer xenografts to epirubicin [18]. Furthermore, demethylation and re-expression of activator protein (AP-2α) is responsible for 5-aza-dC-related increased sensitivity to adriamycin and cisplatin in MDA-MB-231 cancer cells [19]. These results indicate that restoration of the expression of certain hypermethylated genes can increase cancer cell sensitivity to chemotherapeutics. In agreement, when DAC loaded TNPs were delivered into MKN45 cells with sequential application of 5-FU after 72 hours, enhanced anti-proliferative and pro-apoptosis effects were observed in the current study. These enhanced effects were concomitant to TFAP2E upregulation and DKK4 downregulation, as shown with 5-FU resistance in colorectal cancer [3].

Instability constitutes the main shortcoming of DAC, both in cell culture settings [20] and aqueous solutions [21]). Therefore, most *in vitro* studies refresh the culture media with DAC regularly to assess gene expression or methylation status [3, 22]. Moreover, the efficacy of DAC is reduced greatly *in vivo* (half-life 15-25 minutes [23]) because of high hepatic amounts of cytidine deaminase, which rapidly metabolizes cytidine analogues into inactive uridine counterparts [23]. In the present study, we prepared DAC-TNPs to prolong the degradation time of DAC. As shown in Figure 1C, the half-life of encapsulated DAC was increased by three times compared with that of free DAC. Degradation only took 24 hours for completion in the free form, while DAC-NPs and DAC-TNPs remained stable up to 72 hours. The observed effects may be attributed to the advantage of NPs of releasing drugs in a steady continuous pattern. Based on this strategy, DAC-NPs enhanced the DNA demethylation effects of DAC in GC cells, with increased TFAP2E amounts and reduced DKK4 levels. Besides, DAC-TNPs regulated TFAP2E and DKK4 more pronouncedly than DAC-NP, which could be explained by the higher cellular uptake of TNP than NP in MKN45 cells (Figure 3), However, TFAPE upregulation and DKK4 downregulation were not observed in the free DAC group. Additionally, there were no TFAP2E and DKK4 level differences in MKN28 cells (negative control) after application of NPs-DAC and 5-FU (Figure 8), which might explain why there was no statistical difference in the observed inhibitory effects (Figure 5A). Theoretically, NPs could shield DAC from deamination through the EPR effect and gelatinase-stimuli strategy. This may explain why the synergistic anti-tumor effects in *in vivo* studies are more pronounced than *in vitro* (Figure 6).

In conclusion, our strategy of applying 5-FU followed by 72-hour DAC-TNPs treatment efficiently enhanced 5-FU efficacy via demethylation of TFAPE. Therefore, sequential treatment with DAC-TNPs and 5-FU is promising for GC treatment. Since simultaneous treatment with DAC-TNPs and 5-FU showed no effects (data not shown), epigenetic alterations are likely critical for DAC-induced sensitization to chemotherapy drugs. GC cells were first treated with DAC-TNPs for cells to undergo at least 2 divisions before 5-FU administration [24]. The strategy of DAC encapsulation in TNPs has several advantages: (1) enhanced DAC stability, with efficient delivery and cellular uptake because of passive (EPR effect) and active (gelatinases-stimuli) targeting strategies; (2) controlled release of DAC from TNPs, which prolongs DAC effects, which may explain the sensitivity of tumor cells and tumor-bearing xenografts to chemotherapeutic drugs; (3) DAC encapsulation in TNPs that protects from deamination, which could explain why the synergistic antitumor effects *in vivo* study are more pronounced than the *in vitro* ones. However, our *in vivo* study was based on xenograft models, and orthotopic transplantation is needed for further investigation. More experiments are needed in additional cancers in order to further evaluate this sequential treatment strategy. The proposed chemotherapy enhancement of gelatinase-stimuli DAC-TNPs *in vitro* and *in vivo* provides a rationale for the clinical translation of this technology.

## MATERIALS AND METHODS

### Preparation of NPs, gelatinase-stimuli NPs (TNPs), DAC-NPs, and DAC-TNPs

PEG-PCL di-block copolymers and gelatinase-stimuli NPs were obtained by applying previous methods [25]. Drug-loading was based on previously published methods, with a modest modification [26]. In brief, aqueous solutions of DAC (20 mg/ml) were mixed with copolymer (30 mg) in dichloromethane (1 mL) followed by sonication. The mixture was mixed with 5% polyvinyl alcohol (PVA) to generate a double-emulsion, from which dichloromethane was evaporated at room temperature in a fume cupboard. Drug-loaded TNPs and NPs suspension were passed through 1 μm pore membranes (GE Whatman-Xinhua, Shanghai, China); this suspension was lyophilized with pluronic^®^ F68 (40 mg/ml) before storage. Control TNPs and NPs were obtained in a similar fashion without DAC supplementation.

### Physicochemical properties of NPs

NPs were characterized by their size, polydispersity, zeta potential, and morphology. Hydrodynamic size and polydispersity were measured by dynamic light scattering (DLS) (Brookhaven Instruments Corporation, USA). Zeta potential was determined using Zetaplus (Brookhaven Instruments Corporation, USA). The samples were kept at 37°C in phosphate buffered saline (PBS), and their size was monitored for 16 days for stability. The morphology of the NPs was determined by transmission electron microscopy (TEM, JEM-100S, JEOL, Japan). One drop of properly diluted NP suspension was placed on a copper grid covered with nitrocellulose membrane and air-dried at room temperature. The sample was negatively stained with phosphotungstic sodium solution (1% w/v) before observation.

### Drug loading content and encapsulation efficiency

The drug loading and encapsulating process were carried out at 4°C since the decomposition rate of DAC in solution at 5°C is minimal (< 1% for 24 h) [27]. A predetermined amount of freeze-dried NPs were dissolved in the mobile phase, then the suspension was stirred with a magnetic stirrer at 100 rpm for 12 hours under 4°C. The drug loading content (DLC) and encapsulation efficiency (EE) were measured by High Performance Liquid Chromatography (HPLC) system (Agilent 1200 HPLC system, Agilent Technologies, Palo Alto, USA) with C18 reversed phase column (250 mm × 4.6 mm, 5 μM, ZORBAX Eclipse XDB-C18, Agilent Technologies, Palo Alto, USA) as the stationary phase. The mobile phase for DAC included 0.01 M K2HPO4 buffer, pH 6.8, with an injection volume of 20 μL, and a flow rate of 1.0 mL/min. This was held in an isocratic mode for 6 minutes at a wavelength of 220 nm using a UV detector. The retention time of DAC was approximately 5 minutes. The DLC and EE were calculated using the following equations, (1) and (2), respectively:

### DAC degradation experiments

The following steps were carried out in DAC decomposition experiments. Lyophilized TNPs-DAC and NPs-DAC (50 mg) in 2 mL PBS were dialyzed (12 kDa molecular weight cutoff bags; Sigma Aldrich) at 37°C for 72 hours. Samples were collected at various times to assess residual DAC in both DAC-NPs and DAC-TNPs.

### Cell culture

Previous study showed that MKN45 cells are hypermethylated and MKN28 cells hypomethylated [15]. These two cell lines were obtained from Shanghai Institute of Cell Biology (Shanghai, China), and grown in the RPMI 1640 medium containing 10% fetal bovine serum (FBS) at 37°C in a humidified atmosphere with 5% CO2.

### Gelatinase expression and cell uptake assessments

Biocompatibility of NPs was assessed in *in vitro* cellular uptake studies. Gelatinase levels in GC cells were measured by immunohistochemistry; results were confirmed by zymography as previously described [9]. For cellular uptake studies, the nanoparticles were treated with Rhodamine B. MKN45 and MKN28 cells (3 × 10^4^/well) were seeded in 6-well plates and incubated for 24 h, before exposure to Rhodamine B loaded NPs for 2 h. After fixation with 4% paraformaldehyde (PFA) for 30 minutes at room temperature, the cells were assessed for fluorescent signals by fluorescence microscopy (Axio Scope.A1, Zeiss, Germany).

### MTT assay

We utilized the MTT assay to assess the inhibitory effects of NPs as previously described [28]. Cells were plated in 96-well plates at a density of 4000 cells per well and incubated for 24 hours. In short-term experiments, the cells were incubated in presence of DAC (10 μM), DAC-NPs (10 μM DAC eq), or DAC-TNPs (10 μM DAC eq) the day of seeding. After 72 h, cells were further administered 5-FU (20 μg/ml). The groups were defined as follows: saline, empty NPs, empty TNPs, DAC, 5-FU, DAC 72 h+5-FU, DAC-NPs 72 h + 5-FU or DAC-TNPs 72 h + 5-FU. Cell viability rates were assessed by absorbance at 490 nm every 24 h for 3 days.

### *In vivo* antitumor assay

5 × 10^6^ MKN45 cells in 0.1 ml RPMI 1640 medium were subcutaneously injected into right posterior flanks of BALB/c nude mice (male, 4–5 weeks old). Tumor volumes (mm^3^) were derived as W × L^2^/2 (W and L are the width and length, respectively). When 80% of tumors were at least 100 mm^3^, the animals were randomly assigned to treatment cohorts (*n* = 7), treated intravenously with saline control, NPs, TNPs, 5-Fu (20 mg/kg), DAC (15 mg/kg) 72 h + 5-Fu (20 mg/kg), DAC-NPs (15 mg/kg DAC eq,) 72 h + 5-FU (20 mg/kg). DAC-TNPs (15 mg/kg DAC eq) 72 h +5-FU (20 mg/kg). The tumors were measured every 3 days for 21 days after treatment. Data were expressed as relative tumor volumes (100% × V/V0; V and V0 are absolute tumor volume and average group tumor volume at randomization.

### Quantitative real-time reverse-transcription polymerase chain reaction (qRT-PCR)

RT-PCR was performed to assess TFAP2E and DKK4 gene expression levels [3]. Total RNA was extracted TRIzol (Invitrogen, USA) from cells after treatment at indicated times. First strand cDNA was synthesized with Exscrip^TM^ RT reagent Kit (Takara, China). Then, qRT-PCR was carried out with SYBR Green and primers specific for TFAP2E (forward, 5′-TAGACCAGTCCGTG ATCAAGAAAGT-3′; reverse, 5′-AGGTTGAGCCCAATC TTCTC TAAC-3′), DKK4 (forward, 5′-ATATTAGAAA GGCAGCTTGATGAG-3′; reverse, 5′-TTAC AAATTTT CGTCCAAAAATGAC-3′). β-actin was used for normalization (forward, 5′-AGT CGGATACACACA TATTC ATCA-3′; reverse, 5′-ATGGTGGGGTAGATC TTCTTCT-3′). The data were analyzed by the comparative ΔΔCt method.

### Statistical analysis

Data are mean ± SD, and were assessed by Mann-Whitney *U* test. A *P* value below 0.05 indicated statistical significance.

## References

[1] Chen W, Zheng R, Baade PD, Zhang S, Zeng H, Bray F, Jemal A, Yu XQ, He J (2016). Cancer statistics in China, 2015. CA Cancer J Clin.

[2] Oncology TL (2016). Correction to Lancet Oncol.

[3] Ebert MP, Tänzer M, Balluff B, Burgermeister E, Kretzschmar AK, Hughes DJ, Tetzner R, Lofton-Day C, Rosenberg R, Reinacher-Schick AC, Schulmann K, Tannapfel A, Hofheinz R (2012). TFAP2E-DKK4 and chemoresistance in colorectal cancer. N Engl J Med.

[4] Sun J, Du N, Li J, Zhou J, Tao G, Sun S, He J (2016). Transcription Factor AP2epsilon: A Potential Predictor of Chemoresistance in Patients With Gastric Cancer. Technol Cancer Res Treat.

[5] Jabbour E, Issa JP, Garcia-Manero G, Kantarjian H (2008). Evolution of decitabine development: accomplishments, ongoing investigations, and future strategies. Cancer.

[6] Schrump DS, Fischette MR, Nguyen DM, Zhao M, Li X, Kunst TF, Hancox A, Hong JA, Chen GA, Pishchik V, Figg WD, Murgo AJ, Steinberg SM (2006). Phase I study of decitabine-mediated gene expression in patients with cancers involving the lungs, esophagus, or pleura. Clin Cancer Res.

[7] Brown R, Plumb JA (2004). Demethylation of DNA by decitabine in cancer chemotherapy. Expert Rev Anticancer Ther.

[8] Iyer AK, Khaled G, Fang J, Maeda H (2006). Exploiting the enhanced permeability and retention effect for tumor targeting. Drug Discov Today.

[9] Liu Q, Li RT, Qian HQ, Yang M, Zhu ZS, Wu W, Qian XP, Yu LX, Jiang XQ, Liu BR (2012). Gelatinase-stimuli strategy enhances the tumor delivery and therapeutic efficacy of docetaxel-loaded poly(ethylene glycol)-poly(varepsilon-caprolactone) nanoparticles. Int J Nanomedicine.

[10] Vijayaraghavalu S, Labhasetwar V (2013). Efficacy of decitabine-loaded nanogels in overcoming cancer drug resistance is mediated via sustained DNA methyltransferase 1 (DNMT1) depletion. Cancer Lett.

[11] Jung J, Park SJ, Chung HK, Kang HW, Lee SW, Seo MH, Park HJ, Song SY, Jeong SY, Choi EK (2012). Polymeric nanoparticles containing taxanes enhance chemoradiotherapeutic efficacy in non-small cell lung cancer. Int J Radiat Oncol Biol Phys.

[12] McLean L, Soto U, Agama K, Francis J, Jimenez R, Pommier Y, Sowers L, Brantley E (2008). Aminoflavone induces oxidative DNA damage and reactive oxidative species-mediated apoptosis in breast cancer cells. Int J Cancer.

[13] Wang Q, Wu P, Ren W, Xin K, Yang Y, Xie C, Yang C, Liu Q, Yu L, Jiang X, Liu B, Li R, Wang L (2014). Comparative studies of salinomycin-loaded nanoparticles prepared by nanoprecipitation and single emulsion method. Nanoscale Res Lett.

[14] Lu N, Liu Q, Li R, Xie L, Shen J, Guan W, Qian X, Yu L, Ding Y, Jiang X, Liu B (2012). Superior antimetastatic effect of pemetrexed-loaded gelatinase-responsive nanoparticles in a mouse metastasis model. Anticancer Drugs.

[15] Wu FL, Li RT, Yang M, Yue GF, Wang HY, Liu Q, Cui FB, Wu PY, Ding H, Yu LX, Qian XP, Liu BR (2015). Gelatinases-stimuli nanoparticles encapsulating 5-fluorouridine and 5-aza-2′-deoxycytidine enhance the sensitivity of gastric cancer cells to chemical therapeutics. Cancer Lett.

[16] Brown R, Strathdee G (2002). Epigenomics and epigenetic therapy of cancer. Trends Mol Med.

[17] Arnold CN, Goel A, Boland CR (2003). Role of hMLH1 promoter hypermethylation in drug resistance to 5-fluorouracil in colorectal cancer cell lines. Int J Cancer.

[18] Plumb JA, Strathdee G, Sludden J, Kaye SB, Brown R (2000). Reversal of drug resistance in human tumor xenografts by 2′-deoxy-5-azacytidine-induced demethylation of the hMLH1 gene promoter. Cancer Res.

[19] Wajapeyee N, Raut CG, Somasundaram K (2005). Activator protein 2alpha status determines the chemosensitivity of cancer cells: implications in cancer chemotherapy. Cancer Res.

[20] Covey JM, Zaharko DS (1984). Effects of dose and duration of exposure on 5-aza-2′-deoxycytidine cytotoxicity for L1210 leukemia in vitro. Cancer Treat Rep.

[21] Karahoca M, Momparler RL (2013). Pharmacokinetic and pharmacodynamic analysis of 5-aza-2′-deoxycytidine (decitabine) in the design of its dose-schedule for cancer therapy. Clin Epigenetics.

[22] Lirk P, Berger R, Hollmann MW, Fiegl H (2012). Lidocaine time- and dose-dependently demethylates deoxyribonucleic acid in breast cancer cell lines in vitro. Br J Anaesth.

[23] Chabot GG, Bouchard J, Momparler RL (1983). Kinetics of deamination of 5-aza-2′-deoxycytidine and cytosine arabinoside by human liver cytidine deaminase and its inhibition by 3-deazauridine, thymidine or uracil arabinoside. Biochem Pharmacol.

[24] Christman JK (2002). 5-Azacytidine and 5-aza-2′-deoxycytidine as inhibitors of DNA methylation: mechanistic studies and their implications for cancer therapy. Oncogene.

[25] Li R, Li X, Xie L, Ding D, Hu Y, Qian X, Yu L, Ding Y, Jiang X, Liu B (2009). Preparation and evaluation of PEG-PCL nanoparticles for local tetradrine delivery. Int J Pharm.

[26] Liu Q, Li RT, Qian HQ, Wei J, Xie L, Shen J, Yang M, Qian XP, Yu LX, Jiang XQ, Liu BR (2013). Targeted delivery of miR-200c/DOC to inhibit cancer stem cells and cancer cells by the gelatinases-stimuli nanoparticles. Biomaterials.

[27] Jabbour E, Issa JP, Garcia-Manero G, Kantarjian H (2008). Evolution of decitabine development: accomplishments, ongoing investigations, and future strategies. Cancer.

[28] Wu P, Liu Q, Li R, Wang J, Zhen X, Yue G, Wang H, Cui F, Wu F, Yang M, Qian X, Yu L, Jiang X, Liu B (2013). Facile preparation of paclitaxel loaded silk fibroin nanoparticles for enhanced antitumor efficacy by locoregional drug delivery. ACS Appl Mater Interfaces.

